# The Functional Upregulation of Piriform Cortex Is Associated with Cross-Modal Plasticity in Loss of Whisker Tactile Inputs

**DOI:** 10.1371/journal.pone.0041986

**Published:** 2012-08-21

**Authors:** Bing Ye, Li Huang, Zilong Gao, Ping Chen, Hong Ni, Sudong Guan, Yan Zhu, Jin-Hui Wang

**Affiliations:** 1 Department of Physiology, Bengbu Medical College, Bengbu, Anhui, China; 2 State Key Lab for Brain and Cognitive Sciences, Institute of Biophysics, Chinese Academy of Sciences, Beijing, China; Medical College of Georgia, United States of America

## Abstract

**Background:**

Cross-modal plasticity is characterized as the hypersensitivity of remaining modalities after a sensory function is lost in rodents, which ensures their awareness to environmental changes. Cellular and molecular mechanisms underlying cross-modal sensory plasticity remain unclear. We aim to study the role of different types of neurons in cross-modal plasticity.

**Methodology/Principal Findings:**

In addition to behavioral tasks in mice, whole-cell recordings at the excitatory and inhibitory neurons, and their two-photon imaging, were conducted in piriform cortex. We produced a mouse model of cross-modal sensory plasticity that olfactory function was upregulated by trimming whiskers to deprive their sensory inputs. In the meantime of olfactory hypersensitivity, pyramidal neurons and excitatory synapses were functionally upregulated, as well as GABAergic cells and inhibitory synapses were downregulated in piriform cortex from the mice of cross-modal sensory plasticity, compared with controls. A crosswire connection between barrel cortex and piriform cortex was established in cross-modal plasticity.

**Conclusion/Significance:**

An upregulation of pyramidal neurons and a downregulation of GABAergic neurons strengthen the activities of neuronal networks in piriform cortex, which may be responsible for olfactory hypersensitivity after a loss of whisker tactile input. This finding provides the clues for developing therapeutic strategies to promote sensory recovery and substitution.

## Introduction

The loss of a sensation in rodents induces their hypersensitivity to other stimuli. For example, blindness persons showed the enhanced touch and auditory sensations [1], [2], [3], [4], [5], [6], deaf individuals became sensitive to visual input [7], [8], [9], [10], and animals with a loss of olfaction increased their responses to whisker tactile input [11]. Cross-modal sensory plasticity maintains homeostasis in sensory functions and ensures awareness to living environments. The elucidation of the mechanisms underlying cross-modal sensory plasticity is critically important to develop therapeutic strategies for sensory recovery and substitution.

In the cortices for the remaining sensory modalities of cross-modal plasticity, their territories were enlarged [4], [8], [12], neurons were functionally enhanced [11], and neural circuits were rewired [13]. The cell-specific mechanisms underlying the upregulation of the remaining modality cortices remain unclear. If cross-modal plasticity is induced from olfactory deficit to whisker tactile upregulation [11], is its reverse cross-modal plasticity present? We examined whether the deprivation of whisker tactile input induced olfactory hypersensitivity, and how the functional states of GABAergic neurons and pyramidal neurons in piriform cortex were reset for its upregulation during cross-modal sensory plasticity. A balance between pyramidal neurons and GABAergic neurons in piriform cortex toward the excitation is associated with olfactory hypersensitivity after a loss of whisker tactile inputs.

## Results

We reported a mouse model of cross-modal sensory plasticity, in which olfactory deficit led to the upregulation of whisker tactile sensation and the functional change of GABAergic cells in barrel cortex [11]. Here, we aimed to examine its reverse format and cellular mechanisms in piriform cortex. A week after cutting one side of whiskers in mice (Methods), we examined whether loss of whisker tactile inputs upregulated olfactory sensation as well as induced functional changes in the neurons of piriform cortex. Olfactory afferents mainly project to the piriform cortex on ipsilateral side [14], [15], and whisker afferents project to the barrel cortex on contralateral side [16]. The “deprivation” for the periphery was the right side of whiskers, and this term for the central nervous system was the left side of piriform and barrel cortices.

### Loss of whisker tactile input upregulates olfactory function

The sensitivity of the rodents to odors, i.e., the status of olfactory sensation, was evaluated by observing their initial response to odors at a minimal concentration [17], [18], [19]. This minimal concentration can be read out by stimulating animal olfaction with a specific odor in different concentrations. Based on this principle, an olfactometer was designed for observing behavioral response to an odor at threshold concentration to assess olfactory sensitivity [20], [21], [22], [23], [24]. In addition, the principle of air dilution causes odor concentrations to be decreased with distances. This property allows designing a protocol to measure the olfactory function based on the distances between animals and odor source, in which the minimal concentration can be read out when the animals recognize odor source from random toward statistical correction.

**Figure 1:**
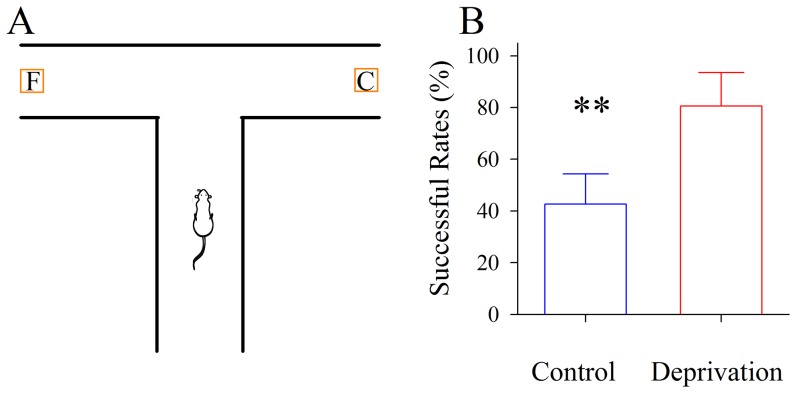
Whisker tactile deprivation upregulates olfactory sensitivity in mice. **A**) shows a “T” maze experiment to identify the olfactory sensitivity in the groups of control and whisker tactile deprivation. A mouse is placed in central arm. A food block (cheese) and a cheese-like block are randomly placed in one side of two arms. **B**) illustrates the statistical analysis for the successful rate of moving into cheese-containing arm versus mouse groups (two asterisks, p<0.001, n = 17).

We used “T” maze to examine the olfactory function of mice (Methods and Fig. 1A). When the odor source (cheese) was moved away from the center of “T” maze, the successful rate for mice moving into the cheese-containing arm was decreased due to the air dilution of cheese odor. If the distance was reached for mice moving into two arms in an equal chance, they were unable to smell cheese. This distance between cheese and “T” maze center was quantified about 40 centimeters on the average in our studies. In this distance, if the successful rate for mice moving into a cheese-containing arm was significantly higher than 50% after the right side of their whiskers was cut, their olfactory sensation was thought to be upregulated, or vice versa.

Fig. 1 illustrates the changes about the olfactory sensation of mice in “T” maze one week after their whiskers are trimmed, i.e., the deprivation of whisker tactile input. Following food deprivation for 12 hours, mice were placed into “T” maze (Fig. 1A), and their motions into either side of two arms were counted. Mice with whisker tactile input deprivation move into a cheese-containing arm with successful rate at 80.6±13% (red bar in Fig. 1B), compared with those control at 42±11.63% (blue; asterisks, p<0.01, n = 17). Olfactory hypersensitivity after cutting mouse whiskers indicates that loss of whisker tactile input upregulates olfactory function, a reverse format of cross-modal sensory plasticity from olfactory deficit to whisker upregulation [11].

To cellular mechanisms underlying this cross-modal plasticity from the loss of whisker tactile input to the upregulation of olfactory sensation, we hypothesized that the functional upregulation of piriform cortex was due to the functional plasticity of pyramidal and GABAergic neurons.

### Loss of whisker tactile input upregulates the functions of pyramidal neurons in piriform cortex

The functions of excitatory pyramidal neurons in piriform cortex were assessed by measuring spike capacity and excitatory synaptic transmission from the mice of controls vs. cross-modal sensory plasticity induced by depriving whisker input. Spontaneous excitatory postsynaptic currents (sEPSC) were recorded on interneurons and pyramidal neurons in piriform cortex under voltage-clamp [25]. The spike capacity (inter-spike intervals, ISI) and active intrinsic properties (threshold potentials, Vts; refractory periods, RP) at the pyramidal neurons were recorded under current-clamp [26], [27], [28]. ISI was measured from sequential spikes induced by depolarization current pulses (150 ms). RPs were measured by injecting multiple depolarization pulses (3 ms and 5% above threshold; Methods) into the neurons after each spike. Threshold potentials were the gaps between resting membrane potential (Vr) and threshold voltage for spiking (Vts).

**Figure 2:**
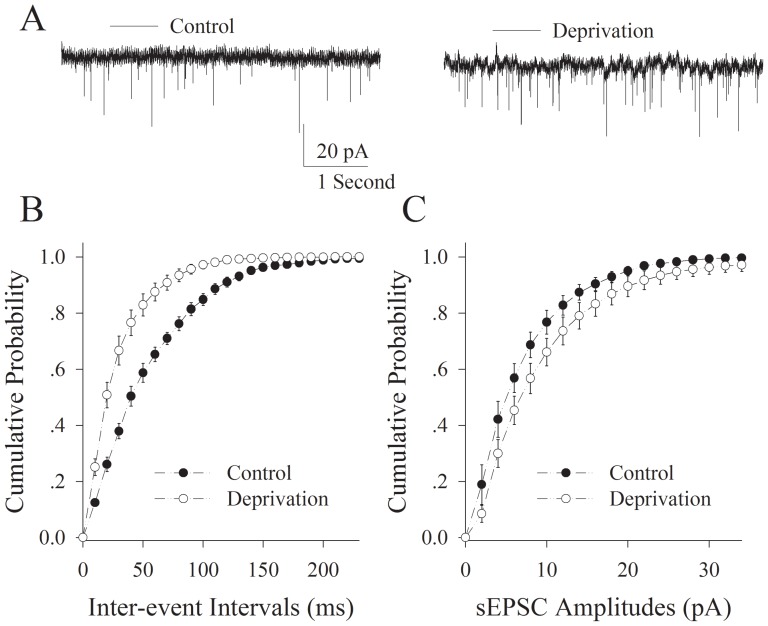
Olfactory upregulation induced by whisker tactile deprivation is associated with an elevated communication from pyramidal neurons to interneurons via excitatory synapses in the piriform cortex. The function of excitatory synapses was evaluated by recording spontaneous excitatory postsynaptic currents (sEPSC) on the interneurons. **A**) shows the recorded sEPSCs on the interneurons in piriform cortex from mice of control (left panel) and whisker tactile deprivation (right). Calibration bars are 15 pA and 1 second. **B**) shows quantitative data about cumulative probability vs. inter-event intervals in controls (filled symbols) and whisker tactile deprivation (hollows; p<0.01, n = 9). **C**) shows cumulative probability versus sEPSC amplitudes in the controls (filled symbols) and whisker tactile deprivation (hollows).

Fig. 2 illustrates the analyses of sEPSCs recorded on the interneurons of piriform cortex from cross-modal plasticity mice versus controls. sEPSC frequency appears higher in cross-modal plasticity mice (right panel in Fig. 2A) than controls (left). Statistical analyses demonstrate shorter inter-event intervals (high sEPSC frequency) in interneurons from cross-modal plasticity mice (open symbols in Fig. 2B) than controls (filled symbols in Fig. 2B; n = 9, p<0.01), but not changes in sEPSC amplitudes (p>0.1, Fig. 2C). As sEPSC frequencies reflect transmitter release [29], [30], [31], this result indicates that the neuronal networks connected via excitatory synapses in the piriform cortex are functionally upregulated in cross-modal plasticity mice induced by depriving their whisker tactile input.

**Figure 3:**
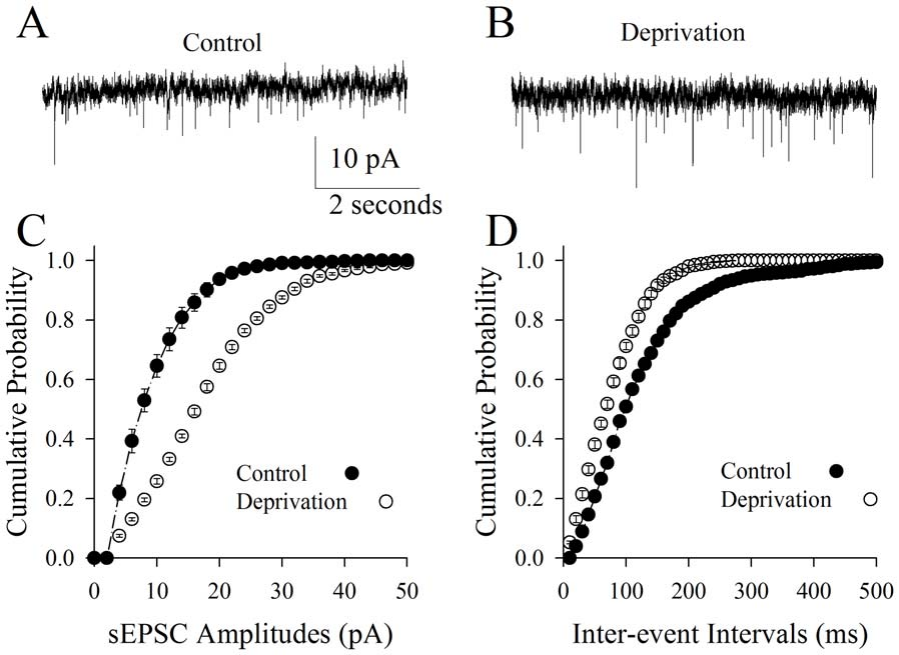
Olfactory upregulation induced by whisker tactile deprivation is associated with an elevated communication among the pyramidal neurons through excitatory synapses in the piriform cortex. The function of excitatory synapses was assessed by recording sEPSC on pyramidal neurons. **A**) shows the recorded sEPSCs on pyramidal neurons in piriform cortex from control mice. **B**) shows the recorded sEPSCs on pyramidal neurons in piriform cortex from mice of whisker tactile deprivation. Calibration bars are 10 pA and 2 second. **C**) shows the quantitative data about cumulative probability vs. sEPSC amplitudes in the control (filled symbols) and whisker tactile deprivation (hollows; p<0.01, n = 8). **D**) illustrates cumulative probability vs. inter-event interval in the control (filled symbols) and whisker tactile deprivation (hollows; p<0.01, n = 8).

sEPSCs were also analyzed on the pyramidal neurons of the piriform cortex from cross-modal plasticity mice and controls (Fig. 3). sEPSC frequency and amplitudes appear higher in cross-modal plasticity mice (Fig. 3B) than controls (3A). Statistical analyses show larger sEPSC amplitudes (Fig. 3C) and shorter inter-event intervals (high sEPSC frequency; Fig. 3D) in the pyramidal neurons from cross-modal plasticity mice (open symbols) than controls (filled symbols; n = 8, p<0.01). In addition to granting the indication above, this result suggests that the sensitivity of pyramidal neurons in piriform cortex to glutamates rises in cross-modal sensory plasticity induced by depriving whisker tactile input. We subsequently examined the changes in the active intrinsic properties of pyramidal neurons.

**Figure 4:**
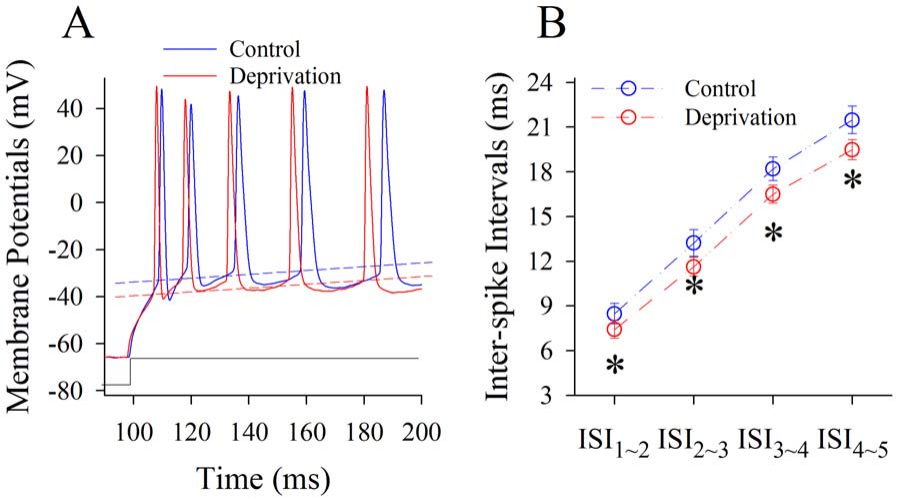
Olfactory upregulation induced by whisker tactile deprivation is associated with an elevated capacity to encode digital spikes at pyramidal neurons of piriform cortex. The depolarization pulses (150 ms, black trace under 4A) were injected into the neurons to evoke sequential spikes. **A**) shows the superimposed waveforms of sequential spikes at pyramidal neurons from a control mouse (blue trace) and a whisker tactile deprivation (red). Dash lines represent threshold potentials. **B**) illustrates quantitative data in inter-spike intervals at pyramidal neurons from control (blue symbols) and from whisker tactile deprivation (red symbols; an asterisk, p<0.05, n = 17).

Fig. 4 shows the effects of whisker tactile input deprivation on sequential spikes at pyramidal neurons in piriform cortex. This manipulation appears to increase the number of spikes in a given time (red trace in Fig. 4A). ISI values for spikes 1∼2 up to 4∼5 are 8.48±0.7, 13.22±0.91, 18.2±0.79 and 21.48±0.92 ms under controls (blue symbols in Fig. 4B); and are 7.43±0.59, 11.63±0.64, 16.5±0.6 and 19.49±0.67 ms under whisker tactile deprivation (reds). ISI values for corresponding spikes under two conditions are statistically different (n = 17, p<0.05). Therefore, the capacity of encoding digital spikes at the pyramidal neurons of piriform cortex is upregulated in cross-modal plasticity mice.

**Figure 5:**
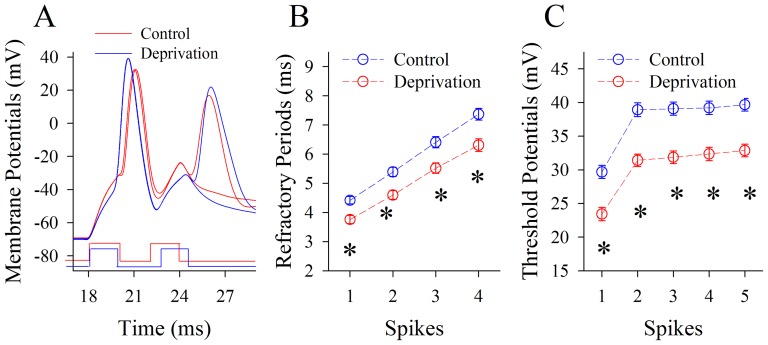
Whisker tactile deprivation attenuates the spike threshold potentials and refractory periods at pyramidal neurons of piriform cortex. The measurement of spikes' threshold potential is showed by dash line in Fig. 4A. **A**) shows the measurement of refractory periods of spike-1 by two pulses at a pyramidal neuron from a control mouse (blue trace) and a whisker tactile deprivation (red trace). **B**) shows the statistical analyses of spikes' refractory periods at pyramidal neurons from controls (blue symbols) and whisker tactile deprivation (red symbols; n = 15, p<0.01). **C**) shows statistical analyses in the threshold potentials of sequential spikes at pyramidal neurons from controls (blue symbols) and whisker tactile deprivation (red symbols; n = 17, p<0.01).

We also studied the influence of whisker tactile input deprivation on Vts and RPs at pyramidal neurons in the piriform cortex (Fig. 5), since these two parameters control the production of sequential spikes. RPs appear shorter in the mice of whisker tactile input deprivation (red traces in Fig. 5A). RP values for spikes 1∼4 are 4.42±0.13, 5.39±0.16, 6.41±0.19 and 7.37±0.2 ms under controls (blue symbols in Fig. 5B); and are 3.76±0.14, 4.6±0.15, 5.52±0.18 and 6.31±0.22 ms under whisker tactile input deprivation (reds). RP values for corresponding spikes are statistically different under these two conditions (n = 15, p<0.01). Moreover, Vts values for spikes 1∼5 are 29.69±0.98, 38.93±1, 39.1±0.97, 39.21±0.98 and 39.68±0.95 mV under controls (blue symbols in Fig. 5C); and Vts are 23.43±0.99, 31.42±0.93, 31.87±0.94, 32.37±0.99 and 32.87±0.94 mV under whisker tactile input deprivation (red symbols). Vts values for corresponding spikes under two conditions are statistically different (p<0.01, n = 17). Thus, a loss of whisker tactile input lowers threshold potentials and shortens refractory periods to upregulate the capacity of encoding digital spikes at pyramidal neurons in piriform cortex.

Taken together, these results suggest that a deprivation of whisker tactile input upregulates the functions of pyramidal neurons and their excitatory networks in piriform cortex (also see Figure S2), leading to olfaction upregulation, i.e., cross-modal sensory plasticity. On the other hand, neural networks are regulated by inhibitory neurons [32], [33], [34], [35], [36], [37], [38], [39], [40]. We further examined the functional changes of GABAergic interneurons in the piriform cortex from the mice of cross-modal plasticity induced by depriving whisker tactile input.

### Loss of whisker tactile input downregulates the function of GABAergic cells in piriform cortex

The functions of GABAergic neurons in the piriform cortex were assessed by measuring their spike capacity and inhibitory synaptic transmission from the mice of control vs. cross-modal sensory plasticity induced by depriving whisker tactile input. The spontaneous inhibitory postsynaptic currents (sIPSC) were recorded on pyramidal neurons in the piriform cortex under voltage-clamp [41]. Inter-spike intervals (ISI) present spike capacity, and their active intrinsic properties include Vts and RPs.

**Figure 6:**
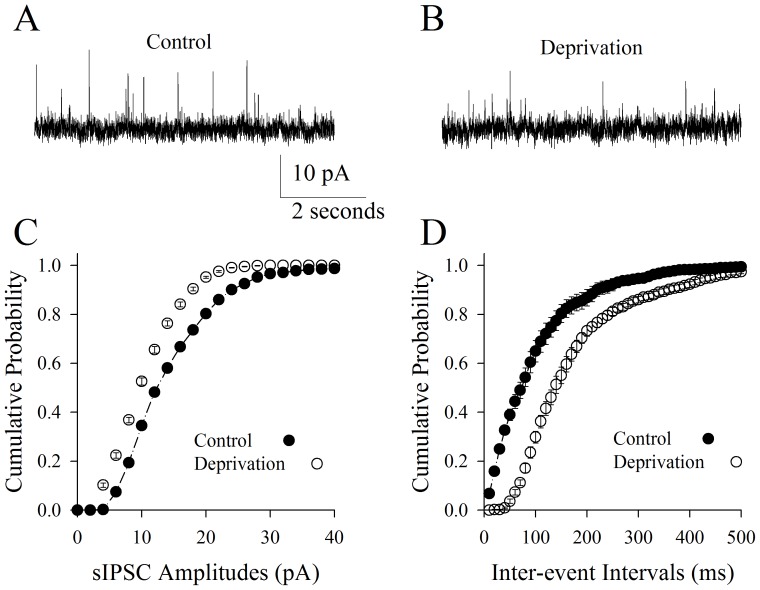
Olfactory upregulation induced by whisker tactile deprivation is associated with a decreased communication from GABAergic neurons to pyramidal neurons via inhibitory synapses in piriform cortex. The function of inhibitory synapses was assessed by recording sIPSC on pyramidal neurons. **A**) shows the recorded sIPSCs on pyramidal neurons in piriform cortex from control mice. **B**) shows the recorded sIPSCs on pyramidal neurons in piriform cortex from mice of whisker tactile deprivation. Calibration bars are 10 pA and 2 second. **C**) shows the quantitative data about cumulative probability vs. sIPSC amplitudes in the control (filled symbols) and whisker tactile deprivation (hollows; p<0.01, n = 10). **D**) illustrates cumulative probability vs. inter-event interval in the control (filled symbols) and whisker tactile deprivation (hollows; p<0.01, n = 10).

Fig. 6 illustrates the analyses of sIPSCs recorded on the pyramidal neurons of piriform cortex from cross-modal plasticity mice vs. control. sIPSC frequency appears lower in cross-modal plasticity mice (Fig. 6B) than control (6A). The statistical analyses show lower sIPSC amplitudes (Fig. 6C) and longer inter-event intervals (low sIPSC frequency; Fig. 6D) in pyramidal neurons from cross-modal plasticity mice (open symbols) than controls (filled symbols; n = 10, p<0.01). This result suggests that inhibitory synapses in piriform cortex are functionally downregulated in cross-modal plasticity mice induced by depriving whisker input. We subsequently examined whether the active intrinsic properties of GABAergic neurons were downregulated in this cross-modal plasticity.

**Figure 7:**
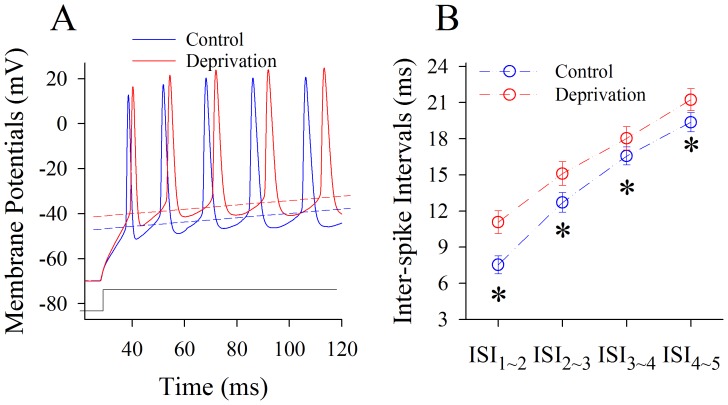
Olfactory upregulation induced by whisker tactile deprivation is associated with a decreased capacity in encoding digital spikes of GABAergic neurons in piriform cortex. Depolarization pulses (150 ms, black trace under 2A) were injected into the neurons to evoke sequential spikes. **A**) shows the superimposed waveforms of sequential spikes at GABAergic neurons from a control mouse (blue trace) and a whisker tactile deprivation (red). Dash lines represent threshold potentials. **B**) illustrates quantitative data in inter-spike intervals at GABAergic neurons from control (blue symbols) and from whisker tactile deprivation (red symbols; an asterisk, p<0.05, n = 18).

Fig. 7 shows the effect of whisker tactile input deprivation on sequential spikes at GABAergic neurons. This manipulation appears to reduce the number of spikes in a given time (Fig. 7A). The ISI values for spikes 1∼2 to 4∼5 are 7.53±0.75, 12.71±0.82, 16.57±0.74 and 19.37±0.8 ms under controls (blue symbols in Fig. 7B); and are 11.1±0.95, 15.11±0.98, 18±0.96 and 21.24±0.91 ms for a week after depriving whisker input (reds). ISI values for corresponding spikes under two conditions are different statistically (n = 18, p<0.05). Therefore, the capacity of encoding digital spikes at GABAergic neurons is downregulated in cross-modal plasticity induced by a loss of whisker tactile inputs.

**Figure 8:**
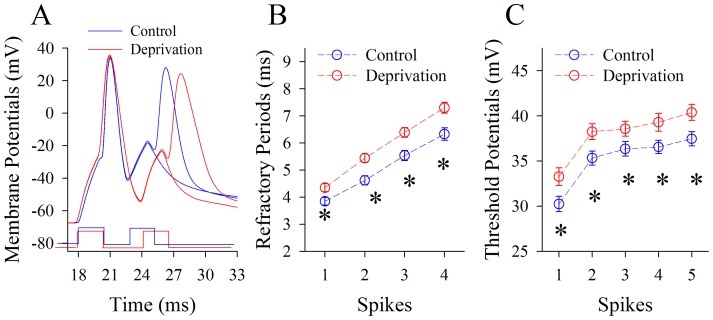
Whisker tactile deprivation elevates the spike threshold potentials and refractory periods at GABAergic neurons of piriform cortex. The measurement of spikes' threshold potential is showed by dash line in Fig. 2A. **A**) shows a measurement of refractory periods of spike-1 by two pulses at a GABAergic neuron from a control mouse (blue trace) and a whisker tactile deprivation (red trace). **B**) shows the statistical analyses of spikes' refractory periods at GABAergic neurons from controls (blue symbols) and whisker tactile deprivation (red symbols; n = 16, p<0.01). **C**) shows statistical analyses in the threshold potentials of sequential spikes at GABAergic neurons from controls (blue symbols) and whisker tactile deprivation (red symbols; n = 18, p<0.01).

Fig. 8 illustrates the effects of whisker tactile input deprivation on Vts and RPs at GABAergic neurons. Their RPs appear to be prolonged (red traces in Fig. 8A). The RP values for spikes 1∼4 are 3.85±0.14, 4.62±0.17, 5.54±0.19 and 6.33±0.23 ms under controls (blue symbols in Fig. 8B); and are 4.35±0.15, 5.44±0.16, 6.39±0.18 and 7.3±0.2 ms under whisker tactile deprivation (reds). RP values for corresponding spikes are statistically different under the two conditions (n = 16, p<0.01). Moreover, the Vts values for spikes 1∼5 are 30.25±0.85, 35.33±0.77, 36.34±0.8, 36.56±0.73 and 37.46±0.78 mV under controls (blue symbols in Fig. 8C); and are 33.27±0.98, 38.25±0.88, 38.57±0.83, 39.3±0.99 and 40.4±0.88 mV under whisker tactile deprivation (reds). Vts values for corresponding spikes under two conditions are statistically different (p<0.01, n = 18). Therefore, a loss of whisker tactile inputs elevates threshold potentials and prolongs refractory periods to downregulate the capacity of encoding digital spikes at GABAergic neurons of piriform cortex.

Briefly, an upregulation of excitatory pyramidal neurons and a downregulation of GABAergic inhibitory neurons in piriform cortex after a loss of whisker tactile input make the function of piriform cortex shifting toward an excitation, which raises its sensitivity to olfactory input. A hypersensitivity of piriform cortex is responsible for an increase of olfactory function in cross-modal sensory plasticity. How is the functional upregulation of piriform cortex initiated after depriving whisker tactile input?

### Crosswire connection between barrel and piriform cortices after deriving whisker tactile input

Our current study indicates that the deprivation of whisker tactile input raises the excitability of the barrel cortex. We proposed that the crosswire connection was established from barrel cortex to piriform cortex, which informed and initiated the piriform cortex to be functional upregulation and be responsible for the increase of olfactory function in this cross-modal sensory plasticity. The functional connection between barrel and piriform cortices was examined by electrophysiology and two-photon cellular imaging in cortical slices.

**Figure 9:**
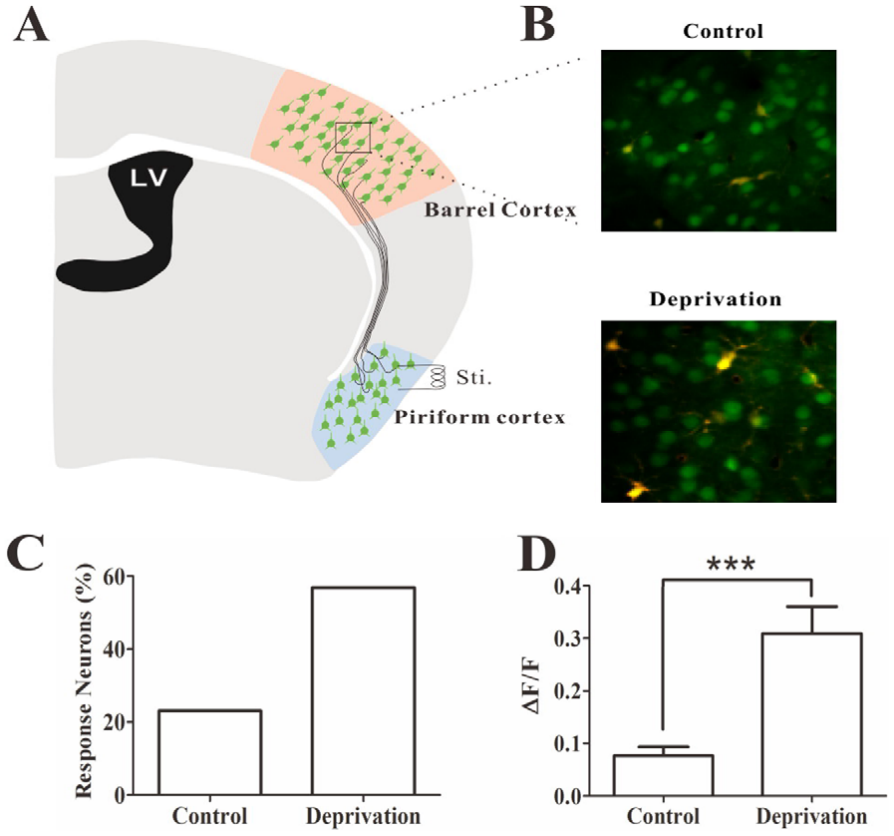
The functional connection is established from the barrel cortex to the piriform cortex during olfactory upregulation induced by depriving whisker tactile input. The activities of network neurons in barrel cortex were recorded by two-photon cellular imaging when the piriform cortex was stimulated electrically. **A**) shows the experimental design, in which neuronal activities in the barrel cortex were recorded under a two-photon microscope and they were activated by stimulating the piriform cortex with a bipolar tungsten electrode. **B**) shows the activity imaging of network neurons in the barrel cortex under the conditions of control (top panel) and whisker input deprivation (bottom). **C**) shows the quantitative data about the percentage of neurons in response to electrical stimulation at piriform cortex under the controls and whisker tactile input deprivation. **D**) illustrates the fluorescent intensity of these neurons is significantly higher in whisker input deprivation mice than control mice (p<0.001, n = 5).

In this study, if network neurons in barrel cortex are able to be activated by antidromic stimuli in piriform cortex, the functional connection is present from the barrel cortex to piriform cortex. Fig. 9A shows experimental design, in which two-photon cellular imaging records the activities of network neurons in barrel cortex and a bipolar tungsten electrode stimulates piriform cortex. Fig. 9B shows the neuronal activities recorded by a two-photon microscope under the conditions of control (top panel) and whisker input deprivation (bottom panel). Compared to the controls, the percentage of responsive neurons is increased after depriving whisker tactile input (Fig. 9C). The fluorescent intensity of these neurons is significantly higher in whisker input deprivation mice than controls (Fig. 9D, p<0.001, n = 5). Thus, the functional connections are established from barrel cortex to piriform cortex in cross-modal sensory plasticity induced by depriving whisker tactile input.

## Discussion

We established a new model of cross-modal sensory plasticity that the loss of whisker tactile input upregulated olfactory sensation in mice (Fig. 1), a reverse format of cross-modal plasticity from olfactory deficit toward whisker upregulation [11]. The reciprocal cross-modal sensory plasticity exists between olfaction and whisker tactile sensation. In terms of cell-specific mechanism in piriform cortex, olfactory hypersensitivity after whiskers are trimmed is associated with an increase in the functions of pyramidal neurons and their network (Figs. 2, 3, 4 and 5) as well as a decrease in the function of GABAergic neurons (Figs. 6, 7 and 8). Such changes lead to functional upregulation in neural networks of piriform cortex and subsequent hypersensitivity in olfaction. The crosswire between barrel cortex and piriform cortex (Fig. 9) may be responsible for the initiation of cross-modal sensory plasticity.

After establishing the cross-modal plasticity that loss of olfaction upregulates tactile sensation [11], we report its reverse format of cross-modal plasticity from whisker tactile input deprivation to olfactory hypersensitivity. Our studies strengthen the concept that cross-modal sensory plasticity can be reciprocally established, such as blind individuals displaying the enhanced auditory function [1], [2], [3], [4], [5], [6], and deaf persons being more alert to visual inputs [7], [8], [9], [10]. In cross-modal sensory plasticity, a hypersensitivity in the remaining sensory modalities and subsequent sensory substitution maintain individuals' awareness to their living environment.

In terms of the mechanisms underlying cross-modal sensory plasticity, the studies indicate the enlargement of cortices for remaining modalities [4], [8], [10], [12], the rewire of neural circuits [13], the upregulation of neuronal function [11] and the elevated expression of certain genes [42], [43]. In studying cell-specific mechanisms, we found that an upregulation of pyramidal neurons and a downregulation of GABAergic neurons in piriform cortex were associated with olfactory sensitization evoked by depriving whisker tactile input, which initiated the crosswire between barrel cortex and piriform cortex and subsequent cross-modal sensory plasticity. These natures for encoding digital spikes in piriform neurons may play critical role in the sensitization of spared sensory modalities in cross-modal plasticity.

After the loss of a sensation, cross-modal sensory plasticity may be initiated by a process that sensory afferents are rewired from the remained sensory organs to the cortical region of losing inputs, which is supported by the following facts. Visual stimuli evoked the potentials in the auditory cortex of deaf individuals, and ring stimuli evoked the potentials in blindness's visual cortex [4], [8], [10], [12], [13]. This rewire of afferents constitutes sensory substitution. In the meantime, the signals to the cortices of the remained modalities may be weakened, which leads to the expansion of these cortices to maintain homeostasis in their sensations and sensory hypersensitivity. This cross-modal projection of afferents can be explained by the repealing among sensory afferents and the attraction of sensory cortices to specific afferents [11]. The signals in Wnts family can repeal and attract the extension of neuronal axons [44], [45], [46].

Potential molecular mechanisms for the rewiring of neural circuits have not been addressed. This rewiring includes the crosswire circuits to be reactivated between sensory cortices as well as the afferents of the remained modalities to project to a sensory cortex that loses input signal. Based on our study, it also needs to be addressed how the neurons are differentially regulated, and how these never cells work coordinately to upregulate olfactory sensation. Our finding in cell-specific mechanism for cross-modal sensory plasticity is an initiative for this topic. It may be questioned whether olfactory upregulation by depriving whisker tactile input on the right side is dominantly present on the left side or right side of olfactory pathway since there is a connection between the two sides of olfactory bulbs [47]. Our study shows a dominant upregulation on the left side of olfactory function as well as of functional changes in piriform cortex.

The sensitivity of rodents to odors was evaluated by testing their initial responses to odors at a minimal concentration [17], [18], [19], so that an olfactometer was designed to observe behavioral responses to odor threshold concentration and to quantify olfaction sensitivity [20], [21], [22], [23], [24]. In fact, air dilution causes odor concentration to be decreased with distances, which grants designing a protocol to measure olfactory sensation based on the distances between animals and odors. Here, we used a “T” maze to examine the olfactory function of mice (Methods and Fig. 1A). When the cheese was moved away from the center of “T” maze, the successful rate for mice moving into cheese-containing arm was decreased. Once a distance is reached, at which mice move into two arms equally, they are unable to smell cheese. Under this distance, if the successful rate for mice moving into a cheese-containing arm is significantly higher than 50%, their olfactory function is upregulated, or vice versa. Our approach to evaluate olfactory sensitivity is based on mice freely searching for food in response to odorants diffused from air dilution gradient, instead of placing them in a restricted space.

A previous report indicated that a functional upregulation of GABAergic neurons in the barrel cortex was associated with cross-modal plasticity induced by depriving olfaction [11]. We also observed that a downregulation of GABAergic neurons in the piriform cortex was associated with the cross-modal plasticity induced by trimming whiskers. The discrepancy between these results may be the followings. The neural circuits in barrel cortex and piriform cortex are different [16], [48], such that the function of GABAergic neurons may be different in regulating neural networks to set activity patterns to be various in these two cortical areas. Second, GABAergic neurons in these two areas respond to the deprivation of sensory inputs differently. The deprivation of olfactory input by impairing olfactory epithelia reduced the number of GABAergic neurons (Ni et al. 2010). The deprivation of whisker tactile input by trimming whiskers did not reduce the number of GABAergic cells and the density of their processes (Figure S1). Third, the approaches to deprive olfactory input and whisker tactile input in these two studies are different. Olfactory input was deprived by impairing olfactory epithelia, i.e., the complete deprivation of olfactory input [11]; whereas whisker tactile input was deprived by trimming whiskers, i.e., the incomplete deprivation of whisker input (Methods). These factors may differently influence cortical functions.

**Figure 10:**
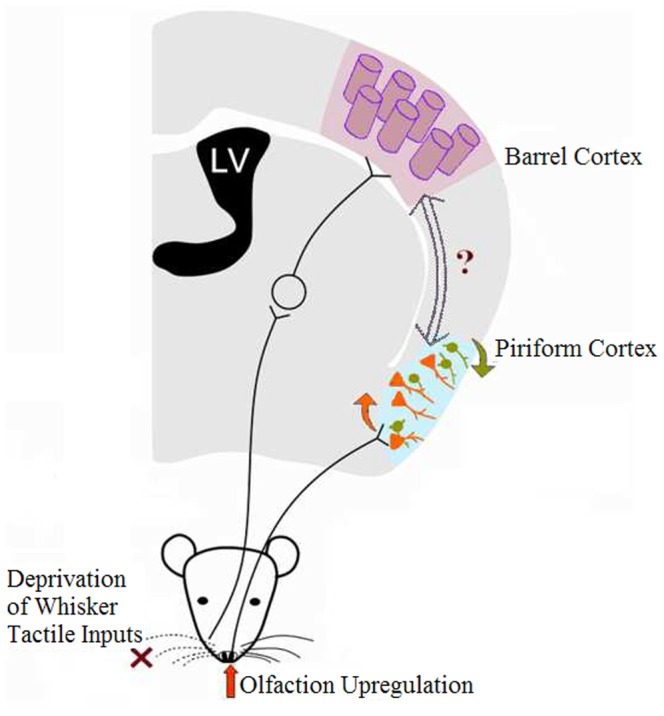
A schematic diagram illustrates cross-modal sensory plasticity from loss of whisker tactile input to olfactory upregulation and the involvement of GABAergic and pyramidal neurons in piriform cortex. The right side of whiskers was cut (the deprivation of whisker tactile inputs, red-cross marker), which leads to the upregulations of olfactory sensitivity (red arrow in front of the nose) and piriform cortex. This cross-modal sensory plasticity from whisker tactile deprivation to olfactory upregulation is accompanied by the increase in the function of pyramidal neurons (orange arrow) and the decrease in the function of GABAergic neurons (green) in the piriform cortex. The information about a loss of whisker tactile inputs from the barrel cortex is transmitted to the piriform cortex via their crosswire connection.

We introduce a new model of cross-modal sensory plasticity from loss of whisker tactile input to olfactory sensitization, a reverse format of whisker upregulation induced by olfactory deficit [11]. Cell-specific mechanisms include a functional upregulation of pyramidal neurons and a functional downregulation of GABAergic cells in piriform cortex, which coordinately strengthens spatial and temporal activities of network neurons to encode olfactory hypersensitivity (Fig. 10). Our data provide avenues for therapeutic treatments to benefit sensory recovery and substitution.

## Methods and Materials

### The establishment of cross-modal plasticity relevant to whisker tactile sensation

The studies and all experiments conducted are fully approved by the Institutional Committee of Animal Care Unit in Administration Office of Laboratory Animals Beijing China (B10831). In the model for the loss of whisker tactile inputs, we cut one side of whiskers in FVB-Tg(GADGFP)4570Swn/J mice (Jackson Lab, USA) whose GABAergic neurons expressed enhanced green fluorescent protein (GFP) and were somatostain-positive [49] at postnatal days 7 and re-cut them every two days to have a closely complete deprivation of whisker input. A group of mice without cutting whiskers was used as a control.

The sensitivity of rodents to odors (the status of olfactory function) is assessed by seeing their initial responses to odors at a minimal concentration [17], [18], [19]. The minimal concentration can be read out by stimulating animal olfactory system with a specific odor in different concentrations. Based on this principle, an olfactometer was designed for observing behavioral response to an odor at threshold concentration to quantify olfactory sensitivity [20], [21], [22], [23], [24]. In addition, the principle of air dilution causes odor concentrations to be decreased with distances. This property allows designing a protocol to measure olfactory sensation based on the distances between animal and odors, in which a minimal concentration can be read out when the animals correctly start responding to the source of odors.

Food-deprived mice search food based on their olfaction. To test their olfactory sensitivity, we placed mice in the central arm of a “T” maze, and the food, such as a piece of cheese, into one side of arms to let them freely searching (Fig. 1A). A cheese-like block was placed on other side of arms as the control to judge the successful rate of mice moving toward the cheese. It is noteworthy that the cheese and cheese-like blocks were randomly placed into both arms. The experiments in all of food- deprived mice for 12 hours were conducted in a quiet red-light room. The olfactory sensitivity was identified by changing the distance of cheese away from the center of “T” maze. The larger is distance, the higher is olfaction sensitivity.

In the experiments with control mice, the distance between cheese and a “T” maze center was changed for mice smelling and searching. If successful rate for mice to move into cheese-containing arm was larger than 50%, this distance from cheese to “T” maze center was in the range where mouse olfaction was able to reach. When the cheese was further moved away from the center of “T” maze, successful rate for these control mice moving into cheese-containing arm was decreased. If a distance was reached at which these mice moved into the two arms equally, they were unable to smell cheese, which was defined as mouse olfactory sensitivity to cheese. This distance between cheese and “T” maze center was about 40 centimeters on the average in our experiments. Under this condition, if the successful rate for mice with whisker tactile input deprivation moving into the cheese-containing arm was significantly higher than 50%, their olfactory function was considered to be upregulated, or vice versa.

One week after whisker tactile input deprivation in mice, we assessed whether their olfaction was upregulated by examining their behaviors that depend on olfactory sensation, such as searching food based on odor smelling in a “T” maze, in which two objects (cheese and cheese-like block) were placed to two sides of arms. The mice were placed at central arm for their searching cheese based on odor smelling. The successful rate above 50% (Results) indicates the upregulation of olfaction.

The cares were also taken in the following issues. The “T” maze was cleaned by 70% ethanol and then wet papers before each trail to remove any odors adhering on the wall of “T” maze. A fresh cheese block was used for each trail to maintain consistency of odor concentration. The experiments were conducted in a 120 m^2^ room with constant ventilation. The movement in the room was limited to prevent making odor plume and noise. Mice moving into either arm above 30 centimeters and staying above 5 seconds were counted as their entry, in which they always touched the cheese in a cheese- containing arm. All mice in two groups were given by one trail during the assessment of olfactory sensitivity to prevent the possibility of their memorizing the simple “T” maze test.

Subsequently, we cut the brain slices from the piriform cortex and studied the active intrinsic properties of pyramidal and GFP-labeled GABAergic neurons by whole-cell recordings to examine cell-specific mechanisms underlying cross-modal plasticity.

### Electrophysiological studies in cortical neurons

The cerebral cortical slices (400 µm) were prepared from FVB-Tg(Gad- GFP)45704Swn/J mice in the groups of controls as well as whisker tactile deprivation with olfactory upregulation. Postnatal days (PND) 18–20 mice were anesthetized by injecting chloral hydrate (300 mg/kg) and decapitated by guillotine. The slices were sectioned with a Vibratome in the modified and oxygenized (95% O_2_ and 5% CO_2_) artificial cerebrospinal fluid (mM: 124 NaCl, 3 KCl, 1.2 NaH_2_PO_4_, 26 NaHCO_3_, 0.5 CaCl_2_, 5 MgSO_4_, 10 dextrose and 5 HEPES; pH 7.35) at 4°C, and were held in normal oxygenated ACSF (mM: 124 NaCl, 3 KCl, 1.2 NaH_2_PO_4_, 26 NaHCO_3_, 2.4 CaCl_2_, 1.3 MgSO_4_, 10 dextrose and 5 HEPES; pH 7.35) 25°C for 1–2 hours. A slice was transferred to the submersion chamber (Warner RC-26G) and perfused with normal ACSF at 31°C for the electrophysiological experiments [11], [50], [51].

GFP-labeled GABAergic neurons and pyramidal neurons in layer II–IV of piriform cortex were recorded by whole-cell voltage- and current-clamp. GABAergic cells appeared round/ovary-like soma and tree branch-like processes under DIC optics (Nikon FN-E600), and were identified under a fluorescent microscopy by excitation wavelength at 488 nm and emission at 525 nm. These neurons demonstrated fast spiking and less adaptation in spike amplitude and frequency, i.e., typical properties for the interneurons [11], [30], [34], [35], [37], [38], [39], [40], [52], [53]. The functional properties of these GABAergic neurons are showed in Figure S3, which are consistent with a previous report [54]. Pyramidal neurons appeared pyramidal in their somata and apical dendrites under DIC optics, and their spikes were characterized as the adaptation in amplitude and frequency, compared with GABAergic neurons.

The functions of excitatory pyramidal neurons in piriform cortex from mice of cross-modal plasticity and control were examined including spontaneous excitatory postsynaptic currents (sEPSC) and spike capacity (inter-spike intervals). sEPSC signals were recorded from pyramidal neurons and GABAergic neurons in slices under voltage-clamp by AxonClamp-200B and inputted into pClamp9 with 100 Hz sampling rate (Axon Instrument Inc., Foster, CA, USA). After the experiments were done, 6-Cyano-7-nitroquinoxaline-2,3-(1*H*,4*H*)-dione (10 µM) and D-amino-5-phosphonovanolenic acid (40 µM) were washed onto the slice to make sure that sEPSCs were glutamatergic. Synaptic efficacy was controlled by presynaptic transmitter release and postsynaptic receptor responsiveness at individual synapses [29], [30], [31], as well as the conversion between inactive synapses and active synapses [52]. In generally, sEPSC frequency shows presynaptic transmitter release, and sEPSC amplitudes do postsynaptic receptor responsiveness and number [25].

Spontaneous inhibitory postsynaptic currents (sIPSC) at GABAergic synapses were recorded by voltage-clamp model (AxonClamp-200B and pClamp9, Axon Instrument, CA, USA) on pyramidal neurons in the piriform cortex. Standard pipette solution contained (mM) 135 K-gluconate, 20 KCl, 4 NaCl, 10 HEPES, 0.5 EGTA, 4 Mg-ATP, and 0.5 Tris–GTP. The osmolarity of pipette solutions was 295–310 mOsmol, and the resistance of filled pipettes was 5∼7 MΩ. Based on Nernst equation, the concentration of chloride ions in this pipette solution makes reversal potential approximately −43 mV, which is consistent with the values in our measurements. Series and input resistances for all of the neurons were monitored by injecting hyperpolarization pulses (5 mV/50 ms) in each experiment, and calculated by voltage pulses vs. instantaneous and steady-state currents. 6-Cyano-7-nitroquinoxaline -2,3-(1*H*,4*H*)-dione (10 µM) and D-amino-5-phosphonovanolenic acid (40 µM) were added into ACSF to block ionotropic receptor-channels in the glutamatergic synapses [41], [55]. This procedure isolated GABAergic IPSCs out. At the end of experiments, bicuculline (10 µM) was washed onto the slices to examine whether synaptic responses were purely mediated by GABA_A_R. Bicuculline did block synaptic currents recorded in our experiments.

Action potentials were recorded under current-clamp at pyramidal neurons and GABAergic neurons in the piriform cortex, which were induced by injecting depolarization pulses. The transient capacitance was compensated, and output bandwidth filter was 3 kHz. The standard pipette solution contained (mM) 150 K-gluconate, 5 NaCl, 0.4 EGTA, 4 Mg-ATP, 4 Na-phosphocreatine, 0.5 Tris- GTP and 10 HEPES (pH 7.4 adjusted by 2M KOH). Fresh pipette solution was filtered by 0.1 µm centrifuge filter before the use. Pipette solution osmolarity was 295–305 mOsmol and the resistance was 6–8 MΩ.

Active intrinsic properties at the neurons include refractory periods after each spike, threshold potentials and spike capacity that is measured by inter-spike interval [11]. Refractory period of spike one (RP1) was measured by injecting two depolarization pulses (3 ms and 5% above threshold) into the neurons, in which pulse one induced spike one at 100% firing probability and inter-pulse intervals were adjusted to have pulse two inducing spike two at 50% firing probability. The duration between spikes 1 and 2 was defined as RP1 [26]. RPs of sequential spikes were measured by multiple depolarization pulses (same as above) into the neurons. Everyone of action potentials, whose RPs were measured, was complete in the amplitude and just out of relative refractory period of its preceding spikes. By adjusting inter-pulse intervals similar to measuring RP1, we read out the durations from complete spikes to their subsequent spikes of 50% firing probability, i.e., the RP of sequential spikes [26], [27]. In order to measure the properties of sequential spikes, the depolarization pulse (150 ms) at an intensity for 10 ms pulse to induce a spike threshold in each of neurons was injected into the neurons to initiate sequential spikes. Inter-spike intervals (ISI) were the duration between the peaks of the neighboring spikes, and threshold potentials (Vts) are the voltages of firing sequential spikes [26], [27], [56]. The correlation of these parameters is presented in our previous studies [57].

### Cellular imaging

Ca^2+^ indicative dye was AM esters of its dye (Oregongreen BAPTA-1-AM) and astrocyte indicator was sulforhodanmine 101-AM (SR101; [58], in which AM element facilitated the dyes to be loaded into brain cells in slices. OGB1-AM was dissolved in DMSO and 20% Pluronic F-127 (2 g Pluronic F-127 in 10 ml DMSO) to have their stock solutions at 1 mM, and then were diluted in the oxygenated ACSF to yield its final concentration at 10 µM. SR-101 was dissolved in distilled water at 1 mM for stock solution and then dissolved in ACSF to 1 µM for final concentration. These dyes in such solutions were loaded into the neurons and astrocytes in amygdala slices were based on a modified method [59]. A slice was placed in an incubation chamber (1 cm in diameter) containing 2 ml of the loading solution at 35°C for 45 min, and the loading solution was then washed out with the oxygenated ACSF. A slice was transferred to a submersion chamber (Warner RC-26G) and perfused by the oxygenated ACSF at 2 ml/min for cellular imaging experiments.

The images of OGB-1 for Ca^2+^ in amygdala neurons and astrocytes and SR-101 for astrocytes were taken by using a two-photon laser scanning microscope (Olympus FV-1000, Olympus, Tokyo Japan). The 2PLSM was equipped by a two-photon laser-beam generator (Mai Tai, Physical Spectrum, USA) and a scanning system mounted onto an upright microscope (Olympus BX61WI) with water immersion objectives (40×, 0.8NA). A laser beam (810 nm) was given to excite OGB-1-AM and SR-101. The emission wave spectra were 523 nm for Ca^2+^-binding OGB-1 and 603 nm for SR-101, respectively. Average power delivered to the brain slices was <10 mW. The parameters set for the laser beam and photomultiplier tube were locked for two groups of slices throughout the experiments in order to have consistent condition in the comparisons of the results between control and whisker input deprivation mice. Images were viewed and analyzed with Fluoviewer. Data are presented as the changes in fluorescence intensity [60].

All fluorescence signals were acquired by using Fluoviewer-10 software (Olympus Inc. Japan) and analyzed from the cell bodies in amygdala. Signals are presented as relative fluorescence change [ΔF/F = (F-F_basal_)/F_basal_] after subtracting background noise from the unstained blood vessels. F is the fluorescence intensity at any time point, and F_basal_ the baseline fluorescence averaged across appointed time course or the whole movie for each cell.

Data were analyzed if the recorded GABAergic neurons had the resting membrane potentials negatively more than −60 mV and action potentials above 95 mV, as well as the recorded pyramidal neurons had resting membrane potential more than −67 mV and action potential above 105 mV. These values were averaged from more than hundreds of neurons in our previous studies to indicate their health status. The criteria for the acceptation of each experiment also included less than 5% changes in the resting membrane potential, spike magnitude, and input/seal resistance. These criteria are based the following facts. The neurons in brain slices are vulnerable to a lack of oxygen and glucose. Their immediate responses to this condition include reducing ATP production and ATPase pump function, and subsequently changing ionic cross-membrane distribution and chemical driving force, which will influence the values of the parameters above. The values of sEPSCs, sIPSCs, inter-spike intervals (ISI, index of spike capacity), Vts, ARPs and Ca^2+^ signals are presented as mean±SE. The comparisons for the data of behavioral tasks, electrophysiology and morphology between groups are done by t-test.

## Supporting Information

Figure S1 **Whisker tactile input deprivation does not induce the changes in the number of GABAergic neurons and the density of their processes in piriform cortex.** GABAergic neurons were genetically labeled with green fluorescent proteins in mice (FVB-Tg(GADGFP)4570Swn/J). **A**) shows an image of GABAergic cells and their process in piriform cortex from a control mouse under a confocal laser scanning microscope. **B**) shows an image of GABAergic neurons and their process in piriform cortex from a mouse of cross-modal sensory plasticity induced by depriving whisker tactile input. **C**) shows statistical analysis for the density of primary processes per GABAergic cell under the conditions of controls and cross-modal plasticity (deprivation; p = 0.065). **D**) illustrates the statistical analysis for the density of secondary processes per GABAergic cell under the conditions of controls and cross-modal plasticity (deprivation; p = 0.41). **E**) shows statistical analysis for the number of GABAergic neurons per field (40× and 512×512 pixels) under controls and cross-modal plasticity (deprivation; p = 0.7).
(DOC)

Figure S2 **Whisker tactile input deprivation induces the increases in the process density of pyramidal neurons and the number of spines per process in piriform cortex.** Pyramidal neurons were genetically labeled with yellow fluorescent protein in mice (B6.Cg-Tg(Thy1-YFPH)2Jrs/J). **A**) shows the images of a pyramidal neuron (left panel) and its dendritic spines (right) in the piriform cortex from a control mouse under a confocal laser scanning microscope. **B**) shows the images of a pyramidal neuron (left panel) and its dendritic spines (right) in the piriform cortex from a mouse of cross-modal sensory plasticity induced by depriving whisker tactile input. **C**) shows statistical analysis for the density of primary processes per pyramidal neuron, mainly basal dendrites, under the conditions of controls and cross-modal plasticity (deprivation; p<0.05). **D**) illustrates the statistical analysis for the density of secondary processes per pyramidal neuron under controls and cross-modal plasticity (deprivation; p = 0.5). **E**) shows statistical analysis for the density of spines per 50 µm process under the conditions of controls and cross-modal plasticity (deprivation; p = 0.4).
(DOC)

Figure S3 **The passive membrane properties of GABAergic neurons in the piriform cortex of mice.** The measurements of passive properties were done by injecting hyperpolarization and depolarization pulses in various intensities. **A**) shows the changes of membrane potentials (top traces) induced by injecting the pulses (bottom) into GABAergic neurons in piriform cortex. **B**) shows the relationship between the injected currents I (pA) and the changes of membrane potentials ΔV (mV), which is linearly correlated (r^2^ = 0.91, p<0.01). **C**) shows the values of the resting membrane potentials in these GABAergic neurons (n = 21).
(DOC)
