# Supplementary material for: The Functional Upregulation of Piriform Cortex Is Associated with Cross-Modal Plasticity in Loss of Whisker Tactile Inputs
Source: PLoS One. 2012 Aug 21;7(8):e41986. doi: 10.1371/journal.pone.0041986 (PMC3424151; doi:10.1371/journal.pone.0041986)
Supplement: Figure S1 — Whisker tactile input deprivation does not induce the changes in the number of GABAergic neurons and the density of their processes in piriform cortex. GABAergic neurons were genetically labeled with green fluorescent proteins in mice (FVB-Tg(GADGFP)4570Swn/J). A) shows an image of GABAergic cells and their process in piriform cortex from a control mouse under a confocal laser scanning microscope. B) shows an image of GABAergic neurons and their process in piriform cortex from a mouse of cross-modal sensory plasticity induced by depriving whisker tactile input. C) shows statistical analysis for the density of primary processes per GABAergic cell under the conditions of controls and cross-modal plasticity (deprivation; p = 0.065). D) illustrates the statistical analysis for the density of secondary processes per GABAergic cell under the conditions of controls and cross-modal plasticity (deprivation; p = 0.41). E) shows statistical analysis for the number of GABAergic neurons per field (40× and 512×512 pixels) under controls and cross-modal plasticity (deprivation; p = 0.7). (DOC) [file pone.0041986.s001.doc]

**
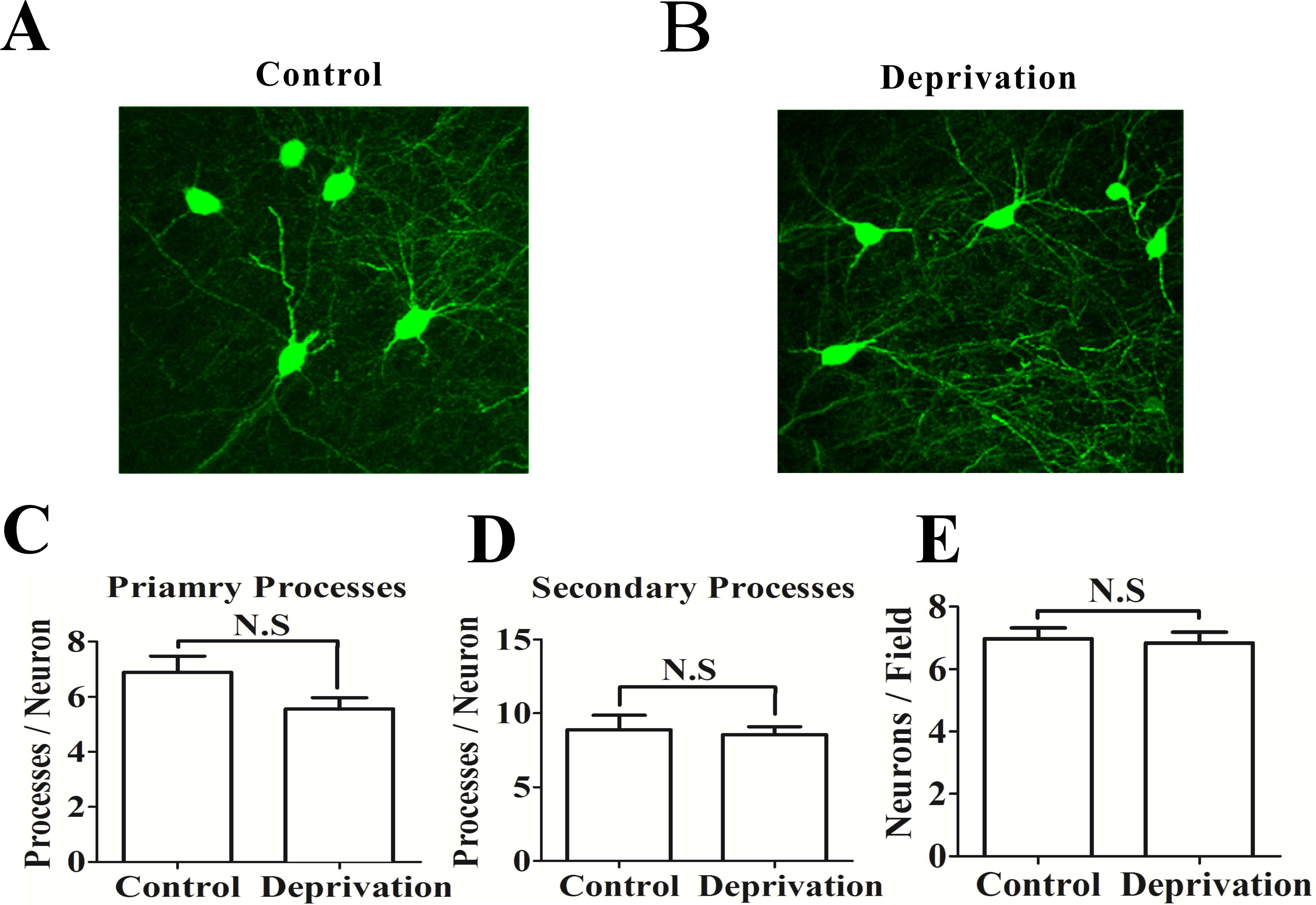
**

**Figure S1** Whisker tactile input deprivation does not induce the changes in the number of GABAergic neurons and the density of their processes in piriform cortex. GABAergic neurons were genetically labeled with green fluorescent proteins in mice (FVB-Tg(GADGFP)4570Swn/J). **A)** shows an image of GABAergic cells and their process in piriform cortex from a control mouse under a confocal laser scanning microscope. **B)** shows an image of GABAergic neurons and their process in piriform cortex from a mouse of cross-modal sensory plasticity induced by depriving whisker tactile input. **C)** shows statistical analysis for the density of primary processes per GABAergic cell under the conditions of controls and cross-modal plasticity (deprivation; p=0.065). **D)** illustrates the statistical analysis for the density of secondary processes per GABAergic cell under the conditions of controls and cross-modal plasticity (deprivation; p=0.41). **E)** shows statistical analysis for the number of GABAergic neurons per field (40X and 512X512 pixels) under controls and cross-modal plasticity (deprivation; p=0.7).
