# Supplementary material for: The Functional Upregulation of Piriform Cortex Is Associated with Cross-Modal Plasticity in Loss of Whisker Tactile Inputs
Source: PLoS One. 2012 Aug 21;7(8):e41986. doi: 10.1371/journal.pone.0041986 (PMC3424151; doi:10.1371/journal.pone.0041986)
Supplement: Figure S2 — Whisker tactile input deprivation induces the increases in the process density of pyramidal neurons and the number of spines per process in piriform cortex. Pyramidal neurons were genetically labeled with yellow fluorescent protein in mice (B6.Cg-Tg(Thy1-YFPH)2Jrs/J). A) shows the images of a pyramidal neuron (left panel) and its dendritic spines (right) in the piriform cortex from a control mouse under a confocal laser scanning microscope. B) shows the images of a pyramidal neuron (left panel) and its dendritic spines (right) in the piriform cortex from a mouse of cross-modal sensory plasticity induced by depriving whisker tactile input. C) shows statistical analysis for the density of primary processes per pyramidal neuron, mainly basal dendrites, under the conditions of controls and cross-modal plasticity (deprivation; p<0.05). D) illustrates the statistical analysis for the density of secondary processes per pyramidal neuron under controls and cross-modal plasticity (deprivation; p = 0.5). E) shows statistical analysis for the density of spines per 50 µm process under the conditions of controls and cross-modal plasticity (deprivation; p = 0.4). (DOC) [file pone.0041986.s002.doc]

**
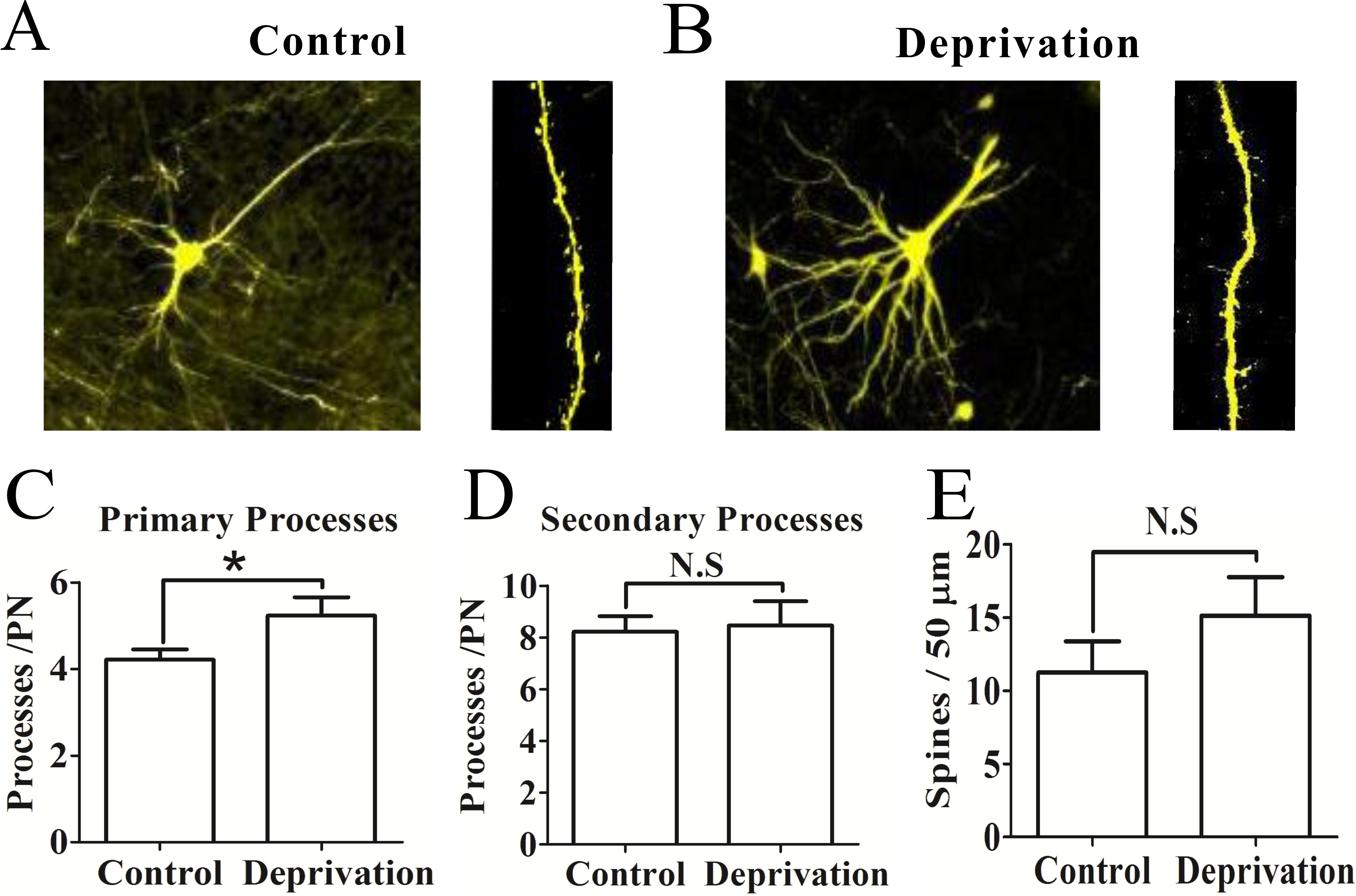
**

**Figure S2** Whisker tactile input deprivation induces the increases in the process density of pyramidal neurons and the number of spines per process in piriform cortex. Pyramidal neurons were genetically labeled with yellow fluorescent protein in mice (B6.Cg-Tg(Thy1-YFPH)2Jrs/J). **A)** shows the images of a pyramidal neuron (left panel) and its dendritic spines (right) in the piriform cortex from a control mouse under a confocal laser scanning microscope. **B)** shows the images of a pyramidal neuron (left panel) and its dendritic spines (right) in the piriform cortex from a mouse of cross-modal sensory plasticity induced by depriving whisker tactile input. **C)** shows statistical analysis for the density of primary processes per pyramidal neuron, mainly basal dendrites, under the conditions of controls and cross-modal plasticity (deprivation; p<0.05). **D)** illustrates the statistical analysis for the density of secondary processes per pyramidal neuron under controls and cross-modal plasticity (deprivation; p=0.5). **E)** shows statistical analysis for the density of spines per 50 μm process under the conditions of controls and cross-modal plasticity (deprivation; p=0.4).
