# Supplementary material for: The Functional Upregulation of Piriform Cortex Is Associated with Cross-Modal Plasticity in Loss of Whisker Tactile Inputs
Source: PLoS One. 2012 Aug 21;7(8):e41986. doi: 10.1371/journal.pone.0041986 (PMC3424151; doi:10.1371/journal.pone.0041986)
Supplement: Figure S3 — The passive membrane properties of GABAergic neurons in the piriform cortex of mice. The measurements of passive properties were done by injecting hyperpolarization and depolarization pulses in various intensities. A) shows the changes of membrane potentials (top traces) induced by injecting the pulses (bottom) into GABAergic neurons in piriform cortex. B) shows the relationship between the injected currents I (pA) and the changes of membrane potentials ΔV (mV), which is linearly correlated (r2 = 0.91, p<0.01). C) shows the values of the resting membrane potentials in these GABAergic neurons (n = 21). (DOC) [file pone.0041986.s003.doc]

**
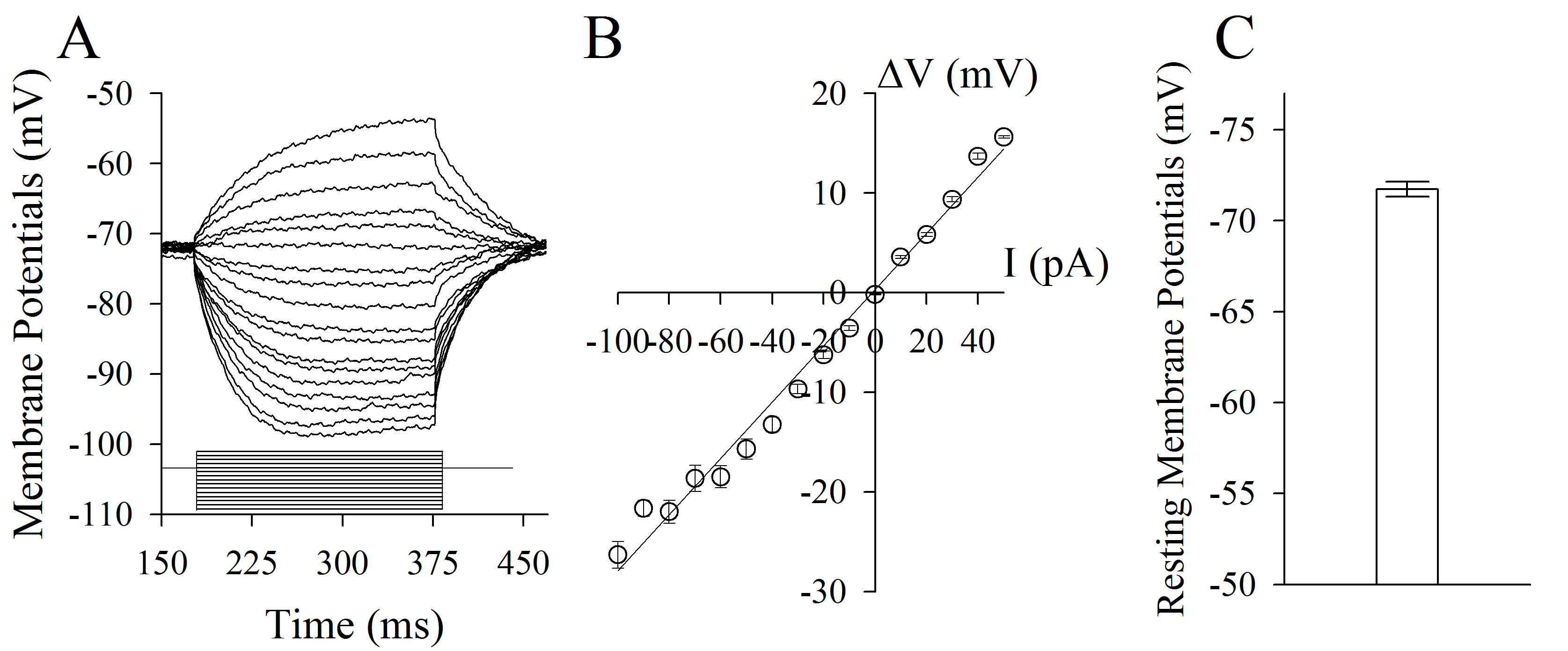
**

**Figure S3** The passive membrane properties of GABAergic neurons in the piriform cortex of mice. The measurements of passive properties were done by injecting hyperpolarization and depolarization pulses in various intensities. **A)** shows the changes of membrane potentials (top traces) induced by injecting the pulses (bottom) into GABAergic neurons in piriform cortex. **B)** shows the relationship between the injected currents I (pA) and the changes of membrane potentials ΔV (mV), which is linearly correlated (r^2^=0.91, p<0.01). **C)** shows the values of the resting membrane potentials in these GABAergic neurons (n=21).
